# Pterygium Pathology: A Prospective Case-Control Study on Tear Film Cytokine Levels

**DOI:** 10.1155/2019/9416262

**Published:** 2019-11-12

**Authors:** Sara I. Van Acker, Michel Haagdorens, Ella Roelant, Jos Rozema, Tine Possemiers, Veerle Van Gerwen, Marie-José Tassignon, Veva De Groot, Sorcha Ní Dhubhghaill, Carina Koppen, Nadia Zakaria

**Affiliations:** ^1^Department of Ophthalmology, Visual Optics and Visual Rehabilitation, University of Antwerp, Wilrijk, ANT, Belgium; ^2^Department of Ophthalmology, Antwerp University Hospital, Edegem, ANT, Belgium; ^3^Clinical Trial Center (CTC), CRC Antwerp, Antwerp University Hospital, University of Antwerp, Edegem, ANT, Belgium

## Abstract

Pterygium is a common eye disease, linked to an increased exposure to UV radiation and dry environments. The associated pathology culminates in visual impairment and, in some rare cases, blindness. However, there remains a lot of uncertainty concerning the pathogenesis of this fibrovascular lesion. As the composition of the tear film provides a reflection into the pathological changes at the ocular surface, tear analysis represents an ideal approach to gain insight in the progression of disease following pterygiectomy. This study enrolled 19 patients and age/gender-matched healthy controls. Tear film levels of interleukin- (IL-) 6, IL-8, and vascular endothelial growth factor (VEGF) were investigated over time, and preoperative concentrations were linked to corneal neovascularization and pterygium size. Diminished tear film levels were found in unilateral patients who show no clinical signs of pterygium recurrence over a period of one year. Hence, our results highlight the potential of using the course of IL-6, IL-8, and VEGF levels in tears as biomarkers for recovery. In addition, when focusing on the affected eyes (i.e., primary and recurrent pterygium), we detected fold changes in preoperative cytokine concentrations to correspond with disease severity. As our proposed biomarkers did not reveal a linear relationship with corneal neovascularization nor the invasive behaviour of pterygium, no exact role in the pterygium pathology could be established. Hence, our data point to these factors being contributors rather than decisive players in the pathological processes.

## 1. Introduction

Pterygium is a common anterior segment disease with a global prevalence of 12% [1]. It is categorised as a benign proliferative lesion or a neoplastic-like growth disorder, owing to the presence of tumour-like characteristics such as altered progenitor cells [2], loss of cell polarity [3], corneal invasiveness and matrix remodelling [4, 5], epithelial cell motility [2, 6], and a high recurrence rate with aberrant proliferation [7]. Ultraviolet (UV) light is thought to trigger its development through limbal epithelial stem cell damage and the upregulation of multiple proinflammatory cytokines, growth factors, and matrix metalloproteinases [8–10]. The standard of care for pterygium currently consists of surgical excision of the affected area, followed by its coverage using a conjunctival autograft or an amniotic membrane [11]. While conjunctival reconstruction is superior to leaving bare sclera, no surgical technique can entirely prevent pterygium recurrence [12]. The reappearance of fibrovascular overgrowth is proposed to be the result of incomplete surgical removal, allowing aberrant or transformed limbal basal cells to reinfiltrate the adjacent conjunctiva and/or limbal epithelium [2]. These recurrence-inducing, infiltrating cells accumulate mutations over time, attributing to their more aggressive proliferative behaviour [13].

Ocular surface inflammation is broadly accepted to play a prominent role in the initial pterygium pathogenesis and in its recurrence [10, 14]. Interestingly, soluble factors contained within tears can provide insight into the pathophysiological state of the ocular surface as they reflect the ongoing intercellular communication [15]. For this purpose, the silicone corneal bath tear sampling technique was developed [16]. Considering proangiogenic cytokine concentrations, our group previously demonstrated that tear film cytokine levels of the corneal tear baths represent a reflection of the localized corneal production and secretion, eliminating any influence by their corresponding serum levels [16].

As the inflammatory process is well-established for aberrant (chronic) wound healing [17] and for tumour development [18], and both conditions show similarities with pterygium [3, 19], it is hypothesized that some of these fundamental proinflammatory communication factors also contribute to the pterygium pathology. Interleukin (IL)-6, IL-8, and vascular endothelial growth factor (VEGF) are prominent players with downstream effects that can be observed in pterygial tissue. Pterygium is characterized by the occurrence of distinct leukocyte populations (i.e., neutrophils, monocytes, mast cells, and T-lymphocytes) [20–22], (lymph)angiogenesis [3, 23], and a hyperplastic epithelium [3]. Both IL-6 and IL-8 promote the chemotactic recruitment of leukocytes [24, 25] and facilitate their in- and efflux by stimulating the development of an extensive lymphatic and vascular network. In contrary to IL-8 signalling [26, 27], IL-6 only indirectly enhances (lymph)angiogenesis through its stimulation of VEGF production [28–30]. Furthermore, IL-8 is known to facilitate epidermal cell division and is therefore likely to contribute to the observed epithelial hyperplasia in pterygium [31].

The balanced IL-6, IL-8, and VEGF tear film secretions in normal subjects [16] are thought to become elevated during pterygium initiation, which is triggered by UVB radiation. UVB has already been described to induce the production of IL-6 and IL-8 in human pterygium epithelial cells [20, 32], human limbal epithelial cells [33], human corneal stromal cells [34], surgically excised pterygia [20], and whole corneas [34]. Despite irradiated limbal epithelial cells and fibroblasts being less potent VEGF producers as compared to their nonirradiated counterparts [35], an increase in VEGF is nonetheless expected as VEGF secretion is stimulated through the UVB-induced IL-6 production. In addition, IL-6 and other UVB-associated proinflammatory secretions contribute to the infiltration of different immune cells that produce proangiogenic factors [35, 36]. Further substantiating our hypothesis of an enhanced tear film cytokine production are the increased IL-6 levels found in reflex tears of pterygium patients [37]. However, to our knowledge, no report has yet been published regarding the course of these proinflammatory cytokine levels after surgical intervention and into the recovery period post pterygiectomy.

Patients suffering from pterygium can sometimes simultaneously be troubled with pinguecula [38]. Pinguecula is a common benign sun-related disorder [38], characterized by the elastotic degeneration of collagen [39] and squamous proliferation and metaplasia [40]. As both the histology and etiology are comparable between pterygium and pinguecula, these conditions are often linked or even mistaken for one another. Their connection is only strengthened as pinguecula has the potential to evolve into pterygium [41]. Hence, eyes with pinguecula should be taken into account when investigating pterygium.

Given the background on IL-6, IL-8, and VEGF, we aimed to quantify their concentrations in the tear film over time and to determine whether these levels could be used to predict pterygium recovery and/or recurrence. In addition, we examined potential correlations between (I) the preoperative (pre-OP) IL-6, IL-8, and VEGF levels; (II) the overall vascularized area of the cornea (%); and (III) the area of the cornea covered by pterygium (%). Age, gender, and lesion grade were considered as possible confounding factors. The tear film concentrations of IL-6, IL-8, and VEGF in eyes with pinguecula (Pi), eyes with primary pterygium (PPt), eyes with recurrent pterygium (RPt), patient's healthy control (PHC) eyes, and healthy control (HC) eyes were compared as well.

## 2. Methods

The study followed the tenets of the Declaration of Helsinki and was approved by the Ethical Committee of the Antwerp University Hospital (EC 11/2/12).

### 2.1. Study Design and Surgical Technique

This was a prospective case-control pilot study, and therefore, no sample size calculations were performed. In total, nineteen patients and age/gender-matched healthy volunteers were enrolled. Informed consent was obtained from all participants before their inclusion. Patients with both unilateral and bilateral pterygium had entered the study, of which five unilateral patients had Pi in their contralateral eye (Table 1). Pi was left untreated, while twenty-one out of twenty-three pterygia were surgically resected. To ensure a representative healthy control group, volunteers had no prior history of ocular pathologies. They did not wear contact lenses nor use eye drops. All pregnant or breastfeeding women were excluded. Slit lamp examination was performed to either confirm the absence of ophthalmic pathologies in control subjects or assist pterygium grading according to the Johnston, Williams, and Sheppard classification system in patients [42]. Under local anaesthesia, the pterygium's head was dissected from the cornea and resected until its base at the Tenon's capsule. A superotemporal autologous conjunctival flap was sutured over the sclerolimbal operating site. Tobramycin/dexamethasone drops (Tobradex, Alcon, Fort Worth, TX, USA) were administered 4 times daily for one and a half month and gradually tapered down over the following 4 weeks. Patients were instructed to maintain lifelong eye lubrication, applying Vidisic® (Bausch & Lomb, Bridgewater, NJ, USA) 4-5 times daily. Bilateral tear samples and ocular surface photographs were obtained from patients prior to pterygium surgery (ranging from 2.5 months preceding until the day of surgery), at 2 weeks (2W-PO), 3 months (3M-PO), and 1 year postoperation (1Y-PO) (Supplementary [Supplementary-material supplementary-material-1]). Bilateral tear samples of the control group were collected. Patients further underwent a thorough clinical examination, including slit lamp examination, at each postoperative visit. In case of recurrence, the RPt was scored using the previously described classification [42]. Patients were contacted again 5 years PO for a final telephonic questionnaire, ascertaining whether pterygium recurrence or other inflammatory pathologies had been diagnosed by their ophthalmologist (Supplementary [Supplementary-material supplementary-material-1]). Moreover, information regarding the use of eye drops and/or other medication was collected.

### 2.2. Tear Samples

All subjects were instructed not to instil ocular medication on the day(s) of tear sampling. Tears were collected at 12 p.m. to avoid diurnal variation of cytokine levels. Sampling and subsequent determination of IL-6, IL-8, and VEGF concentrations were performed as previously described [16]. In brief, the patient was asked to lie in supine position and one drop of topical 0.4% oxybuprocaine hydrochloride was instilled in both eyes. A sterile, silicone rubber cornea bath, encompassing the cornea and limbus, was applied to the sclera, and 1 drop of normal saline was added to the bath. Fifty microliters of the diluted epithelial secretion was immediately picked up using glass capillary micropipettes (1-000-0300, Drummond Scientific, Broomall, PA, USA) and stored in a cryovial at -80°C. The levels of IL-6, IL-8, and VEGF were assessed with a multiplex cytometric bead assay (BD Biosciences Pharmingen, San Diego, CA) according to the manufacturer's instructions. Samples were measured on a FACSArray cytometer (BD Biosciences, San Jose, CA, USA) and analysed using the FCAP Array 1.0.1 software (Soft Flow Inc., Pecs, Hungary).

### 2.3. Patient Photograph Analysis

For each affected eye, the percentage was determined of corneal area covered by pterygium and of corneal neovascularization. Photographs were analysed using ImageJ and an in-house-developed software program based on MATLAB (version 6, MathWorks, Natick, MA, USA), respectively. In ImageJ, the percentage of affected area was determined in triplicate, using the following formula: (affected area on corneal surface/total corneal area)∗100. The average value of the three measurements was used for further analysis. The percentage of corneal neovascularization was analysed as previously described [16]. In brief, the limbus was demarcated on the enface photograph by manually selecting several points on the limbal edge. Specular reflections within this area were then excluded from further processing. In the remaining regions, contrast of blood vessels was enhanced to a maximum and the image was converted to greyscale. Once the converted image corresponded with the vessel distribution in the original picture, the percentage of corneal vascularization due to pterygial encroachment was deduced.

### 2.4. Data Analysis

The three investigated parameters (i.e., tear film levels, percentage of affected corneal area, and percentage of corneal vascularization) are expressed as the mean ± standard error of the mean (SEM). Statistical analysis was performed using the “Statistical Analysis Software” (SAS version 9.4, North Carolina, USA). *p* values lower than 0.05 were considered statistically significant and tear film concentrations below the detection limit are reported as 0 pg/mL. If patients had three tear film levels below this limit, the concentrations were excluded to avoid errors due to sample loss. To correct for measurements coming from the same patient, a linear mixed effect model with a random intercept for subject was fitted. Furthermore, the square root of the outcomes was used to improve model assumptions. When 2 by 2 post hoc comparisons were carried out, a Tukey correction for multiple testing was implemented. In some analyses, only one measurement for each subject was included, thereby allowing us to use the Spearman correlation test (indicated in Results when applied). In addition, the Mann–Whitney *U* test was performed to compare the tear film concentrations pre-OP and 1Y-PO in the affected eye of unilateral patients.

## 3. Results

### 3.1. Patient Demographics

Clinical characteristics of the participants are summarized in Table 1. The average percentage (±SEM) of corneal neovascularization (~vascularization index) and affected corneal area (~area) were calculated for all patients grouped by the different grades (Table 1). In addition, the average tear film concentrations (±SEM) were determined for the five subcategories (Table 1). Overall, only two cases suffered from pterygium recurrence; one patient developed recurrence after 5 months, while pterygium returned after the 1Y-PO consultation in the other patient.

### 3.2. Preoperative Tear Film Levels in Pterygium Subgroups

**Figure 1:**
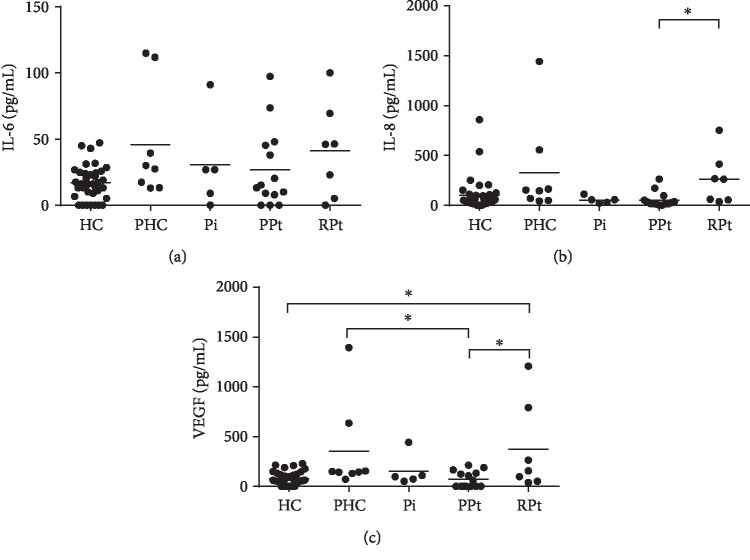
Overview of preoperative IL-6 (a), IL-8 (b), and VEGF (c) concentrations measured in the tear film of healthy controls (HC) and pterygium patients (*n* = 17). Each eye of one patient is regarded as a single entity and divided into one of the four remaining subcategories: patient's unaffected eye or healthy control (PHC), pinguecula (Pi), primary pterygium (PPt), and recurrent pterygium (RPt). Overall, an increase in IL-6 (a), IL-8 (b), and VEGF (c) can be observed in both the RPt and PHC group, with a significantly higher secretion of VEGF (c) being noted between the HC and RPt group. Other significant differences can be found between the PPt and RPt group as well. This includes differences in tear film levels of IL-8 (b) and VEGF (c). The PHC and PPt group also show a dissimilar VEGF (c) concentration. Statistical significance was tested using a linear mixed effect model with 2 by 2 post hoc comparison of the subcategories: ^∗^*p* < 0.05.

PHC and RPt groups showed an overall elevated concentration compared to HC (Figure 1). The difference in VEGF concentration between the HC and RPt groups reached statistical significance (Figure 1(c), post hoc test with the Tukey correction, *p* = 0.011). Contrary to the expectation, the average IL-8 and VEGF concentrations in patients with PPt were lower than the average concentrations seen in the HC group (Figures 1(b) and 1(c)). Similarly, reduced IL-6, IL-8, and VEGF tear film concentrations were observed in eyes with PPt when set against PHC eyes with VEGF levels indicating a significant difference (Figure 1, post hoc test with the Tukey correction, *p* = 0.016). Tear films obtained from patient's eyes with RPt had inflammatory IL-6, IL-8, and VEGF levels surpassing those found in Pi and PPt (Figure 1, IL-8 (Figure 1(b)) and VEGF (Figure 1(c)): *p* < 0.05 for PPt vs. RPt). The Pi and PPt group showed similar interleukin concentrations, while the VEGF concentration was more than twice as high in the Pi group as compared to the PPt group, albeit nonsignificant (Figure 1).

### 3.3. Association Analysis of Clinical Parameters

**Figure 2:**
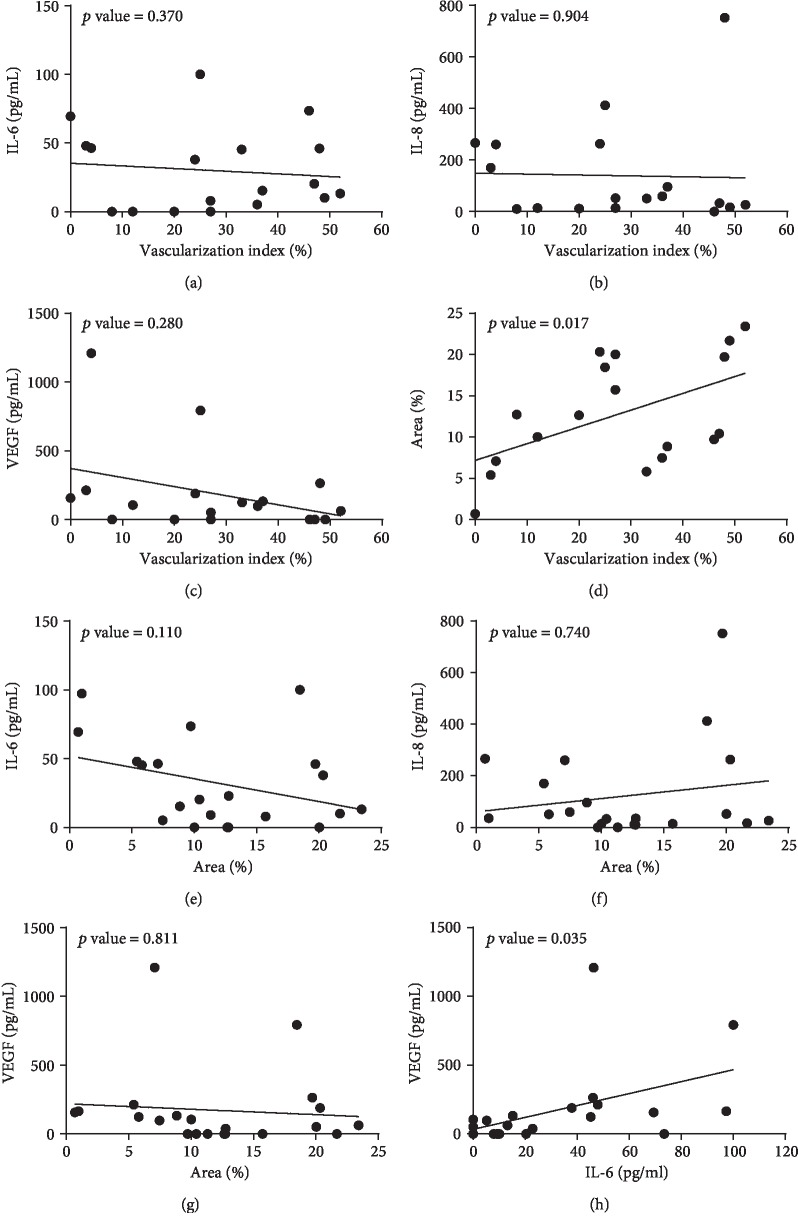
Overview of the association analysis between (I) the overall vascularized area on the ocular surface (~vascularization index) and the preoperative IL-6 (a), IL-8 (b), and VEGF (c) tear film levels, (II) the vascularization index and the area on the cornea covered by pterygium (~area) (d), (III) the area and preoperative IL-6 (e), IL-8 (f), and VEGF (g) levels, and (IV) the preoperative IL-6 and VEGF (h) tear film levels in pterygium patients (*n* eyes = 21, PPt+RPt). Out of the eight analyses, only two combinations revealed a linear positive relationship, i.e., between vascularization index and area (d) and between IL-6 and VEGF (h). Statistical significance was tested using a linear mixed effect model, and the corresponding *p* values are depicted on the graphs. For illustrative purposes, a simple linear regression line is represented.

The relationship between (I) the pre-OP cytokine production (IL-6, IL-8, and VEGF), (II) the vascularization index, and (III) the area which was determined in patients with PPt (Supplementary [Supplementary-material supplementary-material-1]) and RPt (Supplementary [Supplementary-material supplementary-material-1]) and all pterygium patients as a group (*n* eyes = 21, PPt+RPt, Figure 2). Whenever it was unclear which variable should be predictor or outcome in the mixed effect model, an arbitrary choice was made, as we verified that switching the variables as predictor/outcome did not lead to different conclusions. In the complete pterygium group, a clear association was observed between the vascularization index and the area (Figure 2(d), *p* = 0.017) and between the IL-6 and VEGF tear film concentrations (Figure 2(h), *p* = 0.035). The latter also showed a trend towards significance in PPt (Supplementary [Supplementary-material supplementary-material-1], *p* = 0.071) and RPt (Supplementary [Supplementary-material supplementary-material-1], Spearman's correlation, *ρ* = 0.71, *p* = 0.071). Furthermore, a significant linear relationship was revealed between IL-6 levels and area (Supplementary [Supplementary-material supplementary-material-1], *p* = 0.042), however, only in the PPt group. Of note, the *p* values of all other associations exceeded 0.10.

To exclude the possibility of confounding factors, the influence of age, gender, and pterygium grade on the investigated variables was determined. No presumable evidence was found that these variables should be treated as such.

### 3.4. Tear Film Cytokines and Growth Factor: Course over Time

**Figure 3:**
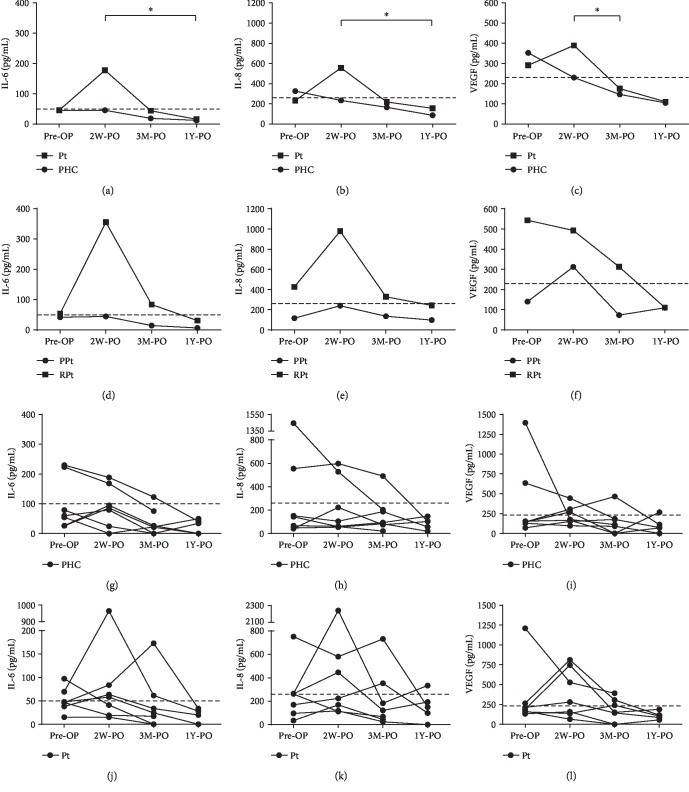
Concentrations of IL-6 (a, d, g, j), IL-8 (b, e, h, k), and VEGF (c, f, i, l) measured in the tear film of unilateral patients at four different time points, i.e., preoperative (pre-OP), two weeks postoperation (2W-PO), three months postoperation (3M-PO), and one year postoperation (1Y-PO). The dotted line in all the graphs represents the upper limit of IL-6, IL-8, and VEGF concentrations in the tear film of healthy controls (HC). Statistical significance was tested using a linear mixed effect model with 2 by 2 post hoc comparisons of the different time points: ^∗^*p* < 0.05. One patient-specific course is not depicted due to missing values. Abbreviations: Pt: pterygium affected; PHC: patient healthy control; PPt: primary pterygium; RPt: recurrent pterygium.

The potential application of IL-6, IL-8, and VEGF concentrations to predict the outcome of pterygium surgery was based on their evolution over time. As only two patients experienced recurrence, the use of IL-6, IL-8, and VEGF course was solely explored as biomarker for recovery and not for recurrence. Unilateral patients without Pi (*n* = 8) were exclusively included in this analysis due to emerging evidence that both eyes can react to a pathological stimulus solely present in one eye [16]. An arbitrary threshold was set (Figure 3), representing an upper limit that encompassed almost all measured IL-6, IL-8, and VEGF concentrations in the HC group (IL-6, 50 ng/mL; IL-8, 260 ng/mL; VEGF, 230 ng/mL).

When fitting a linear mixed effect model, no significant interaction was observed between time and health status of the eye (IL-6, *p* = 0.582; IL-8, *p* = 0.437; VEGF, *p* = 0.696). Despite that the evolution over time did not differ in the PHC and affected eyes (PPt and RPt), the concentrations did significantly vary over the study period (mixed effect model without interaction term; IL-6, *p* = 0.012; IL-8, *p* = 0.047; VEGF, *p* = 0.012) (Figures 3(a)–3(c)). A downward trend was observed in the PHC group when looking at the overall course of the tear film concentrations (Figures 3(a)–3(c)). In spite of the average PHC concentrations being higher than those of HC (Figure 1), most of the concentrations did lie within the HC threshold (Figures 3(g)–3(i)). The course of the average concentrations in the affected eyes showed an elevation at 2W-PO, which was accompanied by a decrease below the HC threshold at the 3M-PO time point (Figures 3(a)–3(c)). These 2W-PO elevations significantly differed from later time points for the three analytes (Figures 3(a)–3(c)). At 1Y-PO, the tear film levels were compared with those of HC and no significant differences were noticed (Mann–Whitney *U* test; IL-6, *p* = 0.975; IL-8, *p* = 0.185; VEGF, *p* = 0.234).

The Pt group was further divided into PPt (*n* = 5) and RPt (*n* = 3) groups to assess if the IL-6, IL-8, and VEGF course was similar over time (Figures 3(d)–3(f)). Bearing in mind that the sample size is rather small, we did not find evidence of a different time evolution for PPt and RPt. In the linear mixed effect model without the interaction, the VEGF (and IL-8) concentrations were detected to vary over the study period as well (time effect: VEGF, *p* = 0.039; IL-8, *p* = 0.079). Furthermore, higher values in the RPt group (Figure 1) were also observed in the following time points (Figures 3(d)–3(f)). The concentration difference between PPt and RPt showed a trend towards statistical significance for the two interleukins: IL-6 (*p* = 0.089) and IL-8 (*p* = 0.091).

## 4. Discussion

Tear fluid represents an ideal source for prognostic biomarkers as the collection is noninvasive, simple, safe, and close to the disease pathology [43]. The encouraging potential of tear-based analyses and their clinical implementation is reflected by the recent development of diagnostic kits, e.g., the detection of lactoferrin and IgE in tear films for the diagnosis of dry eyes and ocular allergies (Advanced Tear Diagnostics, Birmingham). As inflammation plays a pivotal role in the aetiopathogenesis of pterygium, we aimed to elucidate the outcome of pterygiectomy by investigating the potential of IL-6, IL-8, and VEGF as biomarkers for recovery. In addition, to have a better understanding of the role of these cytokines in pterygium pathology, we quantified tear film levels in an HC group and in four patient subcategories (i.e., PHC, Pi, PPt, and RPt). Values were further linked to the degree of neovascularization and to pterygium size as to reveal any potential correlation.

Some surprising differences were observed when comparing the pre-OP IL-6, IL-8, and VEGF concentrations in the HC, PHC, Pi, PPt, and RPt groups. Against expectations, the highest IL-6 and IL-8 concentrations were not detected in the affected eyes (PPt and RPt), but rather in the PHC eyes. In an endeavour to interpret this observation, we will first focus on the general increase in tear film levels in both eyes and afterwards on the level difference in the patient's healthy and affected eye. Of note, the pre-OP levels in the affected eyes do exceed the corresponding concentration measured 1Y after surgery (Figures 3(j)–3(l)), indicating that the levels are considered elevated with respect to the patient-specific healthy values. As both eyes are exposed to UV radiation, it is likely that an upregulation of proinflammatory cytokines takes place at each side. However, at one point, chronic UV radiation combined with the consequences of other demographic, environmental, and life factors [1] introduces enough alterations to initiate pterygium development in one or even both eyes. It is understood that the levels and interplay between proinflammatory cytokines undergo a shift during the progression from chronic UV radiation towards pterygium initiation and development. Moreover, the (yet unknown) combination of these specific proinflammatory cytokines is thought to have a more detrimental effect than just a higher fold change in general proinflammatory players. Therefore, before pterygium resection, it seems that the exact fold change increase of the investigated proinflammatory cytokines does not correlate with the health status of the patient's eyes (PHC vs. pterygium). Furthermore, in spite of the pre-OP levels of IL-8 and VEGF considered elevated in PPt (Figures 3(e) and 3(f), comparison with 1Y-PO levels), they are lower than those observed in HC (Figures 1(b), 1(c), 3(k), and 3(l)). This finding could be attributed to a large interindividual variability in unstimulated basal secretions [15], in cytokine response [44, 45], and in our small sample size. In consonance with the more aggressive behaviour of RPt [46], we found the levels of IL-6, IL-8, and VEGF to be significantly higher than those observed in Pi and PPt.

Next, we turned to analysing the course of the prominent proinflammatory players in the patient's eyes. Our data show that, in the affected eyes, the subsequent decrease of the IL-6, IL-8, and VEGF following the 2W-PO elevation corresponds well with the recovery status of the pterygium patients. The 2W-PO itself is probably attributable to the surgically induced inflammation and wound healing process [47]. No insight into the health status of the eye can be obtained through the comparison of the patient's courses with the average HC levels as a considerable part of the patient's person-specific concentration lies within the HC range (Figures 3(j)–3(l)). In addition, no straightforward relation can be established between the course of the PHC eye and the ocular surface condition. Patients with elevated tear film levels in their healthy eye at the different investigated time points did not develop a clinical inflammatory disease phenotype up to four years after the last sample collection. An analogy could be drawn with inflammatory bowel disease, where an elevation of proinflammatory cytokines in histologically unaffected tissue is not enough to trigger mucosal damage or tissue pathology on itself [48]. Taken together, only the courses of IL-6, IL-8, and VEGF are of predictive value and this solely in the patient's affected eye (PPt and RPt).

Comparing our data to what has been described previously in the literature is challenging, given the variances in the used sampling methods and given the array of different types of inflammatory ocular pathologies that have been included in studies (supplementary [Supplementary-material supplementary-material-1], pre-OP levels). The diversity and observed differences between similar methods encourage the use of meta-analyses and the inclusion of larger cohorts in forthcoming studies.

Our association analysis between the clinical parameters supports the current understanding of IL-6 being a stimulant for the synthesis and release of VEGF [49, 50]. Furthermore, this study provides evidence for a more aggressive and invasive behaviour (% corneal area affected by pterygium) being correlated with a more inflamed and vascularized stroma (vascularization index). This finding was already established for recurrent pterygium [46], but can now be broadened to include all cases of pterygium.

Although we were able to distinguish some significant associations, differences in tear film levels between subgroups, and changes over time, we acknowledge the limitations of a small sample size and its influence on the statistical power. Hence, a larger study population could give us more insight in the different associations involved as well as in more subtle differences in tear film levels between subgroups and in their changes over time. Another subject matter that should be addressed is the gradual tapering of tobramycin/dexamethasone administration over a period of 10 weeks after surgery. The use of these drops is part of the standard-of-care protocol at the Antwerp University Hospital. Dexamethasone belongs to the class of glucocorticosteroids, commonly used after corneal injury and eye surgery. Such therapy effectively reduces ocular inflammation by diminishing the expression of inflammatory cytokines and of MMP as shown in an alkali corneal burn mouse model [51]. As the drops are solely applied in the operated eye after surgery, a potential influence on the tear film levels in the contralateral eye can be excluded as well as a possible influence on the preoperative and 1Y postoperative values. However, we acknowledge that tobramycin/dexamethasone could affect the 2W-PO and potentially the 3M-PO concentrations. The washout period is often considered to be one month [52, 53], making the effect of glucocorticosteroids administration to be possibly present at the 3M-PO time point, though to a lower extent. The overall pharmacological effect is nonetheless considered to be inferior to the resection of the diseased tissue as the tear film levels do correlate with pterygium recovery and do not show significant changes when the medication is ceased.

## 5. Conclusions

We established a role for IL-6, IL-8, and VEGF in pterygium pathology, manifesting as a clear change and an eventual decrease in tear film levels one year after the surgery. As the observed course correlates with the absence of recurrence in unilateral patients, it could be used as a biomarker for recovery. The fold change of concentrations in the affected eyes seems to correspond with the disease severity as RPt patients show higher average levels at different time points compared to those affected by PPt. The same analogy can, however, not be applied to the patient's healthy eye and affected eye. Furthermore, results of the association analysis show a linear relationship between IL-6 and VEGF, strengthening the hypothesis that IL-6 signalling stimulates VEGF secretion in pterygium.

Nonetheless, the lack of relationship between IL-6/VEGF and the vascularization index indicates that IL-6 and VEGF are not decisive factors in pterygium pathology. Neovascularization is in all probability a multifaceted process, consistent with the invasive behaviour of pterygium. As an association is observed between corneal neovascularization and invasiveness (linked to pterygium size), we hypothesize that these processes depend on similar cellular signals and pathways.

## Figures and Tables

**Table 1:** Patient demographics.

Total number of patients	
Male	11
Female	8
Age of patients (years)	
Mean ± SEM	52 ± 4
Range	23-78
Subgroups^∗^	
Pinguecula	5
Primary	15
Recurrent	8
PHC	10
HC	37
Side of involvement	
Left	6
Right	9
Bilateral	4
Classification^∗^	
I+II	3
III	18
IV	2
Vascularization index^∗^^,†^ (%)	
All patients (*n* = 19)	26.9 ± 3.9
Grades I+II (*n* = 2)	6.5 ± 6.5
Grade III (*n* = 14)	28.4 ± 4.5
Grade IV (*n* = 3)	33.7 ± 9.1
Affected corneal area^∗^ (%)	
All patients (*n* = 23)	11.8 ± 1.3
Grades I+II (*n* = 3)	2.4 ± 1.6
Grade III (*n* = 17)	11.9 ± 1.1
Grade IV (*n* = 3)	20.7 ± 1.4
IL-6 tear film levels^∗^^,‡^ (pg/mL)	
Pinguecula (*n* = 5)	30.8 ± 16.0
Primary (*n* = 14)	27.0 ± 8.0
Recurrent (*n* = 7)	41.5 ± 13.5
PHC (*n* = 8)	45.9 ± 15.1
HC (*n* = 37)	17.0 ± 2.1
IL-8 tear film levels^∗^^,‡^ (pg/mL)	
Pinguecula (*n* = 5)	53.3 ± 15.8
Primary (*n* = 14)	53.1 ± 20.2
Recurrent (*n* = 7)	262.6 ± 97.5
PHC (*n* = 8)	326.9 ± 169.9
HC (*n* = 37)	99.0 ± 26.6
VEGF tear film levels^∗^^,‡^ (pg/mL)	
Pinguecula (*n* = 5)	154.9 ± 72.6
Primary (*n* = 14)	71.1 ± 21.8
Recurrent (*n* = 7)	373.1 ± 170.8
PHC (*n* = 8)	352.6 ± 161.8
HC (*n* = 37)	78.1 ± 11.0

The mean ± standard error of the mean (SEM) of each group is given for the following parameters: age, vascularization index, affected corneal area, and IL-6, IL-8, and VEGF tear film levels. ^∗^*n* = 1 = one eye. ^†^The vascularization index could not be calculated for each patient due to the influence of the iris colour and undesirable shadows on the ocular surface photographs. ^‡^Two patients are excluded from the cytokine analysis. One patient suffered from multiple myeloma, involving IL-6 and IL-8 in its pathogenesis. As serum derivatives can passively leak into the tear film, the corresponding concentrations are considered unreliable. The other patient has been excluded due to the presence of two pterygia in one eye, of which the second pterygium has not been surgically removed. PHC: patient's healthy eye; HC: healthy control.

## Data Availability

The data used to support the findings of this study are available from the corresponding author upon request.

## Supplementary Materials

Supplementary 1 Supplementary figure S1: the study protocol on the data collection and patient follow-up. To address our research questions, the patients underwent a medical consultation before the operation (pre-OP, ranging from 2.5 months preceding until the day of surgery), at 2 weeks postoperation (2W-PO), 3 months PO (3M-PO), and 1 year PO (1Y-PO). Bilateral tear samples and ocular surface photographs were taken at each visit. The final follow-up consultation was scheduled 5 years after the surgery (5Y-PO) and consisted of a telephonic questionnaire.

Supplementary 2 Supplementary figure S2: an overview of the association analysis between the preoperative (I) IL-6, IL-8, and VEGF tear film levels; (II) the overall vascularized area on the ocular surface (~vascularization index); and (III) the area on the cornea covered by pterygium (~area) in primary (S2A-H) and recurrent pterygium patients (S2I-P).

Supplementary 3 Supplementary table S3: the reported IL-6, IL-8, and VEGF tear film levels and the corresponding sampling method (i.e., tear type, location, and volume) in healthy individuals and patients with inflammatory ocular surface diseases.
