# Supplementary figures and images for: Pterygium Pathology: A Prospective Case-Control Study on Tear Film Cytokine Levels
Source: Mediators Inflamm. 2019 Nov 12;2019:9416262. doi: 10.1155/2019/9416262 (PMC6875004; doi:10.1155/2019/9416262)

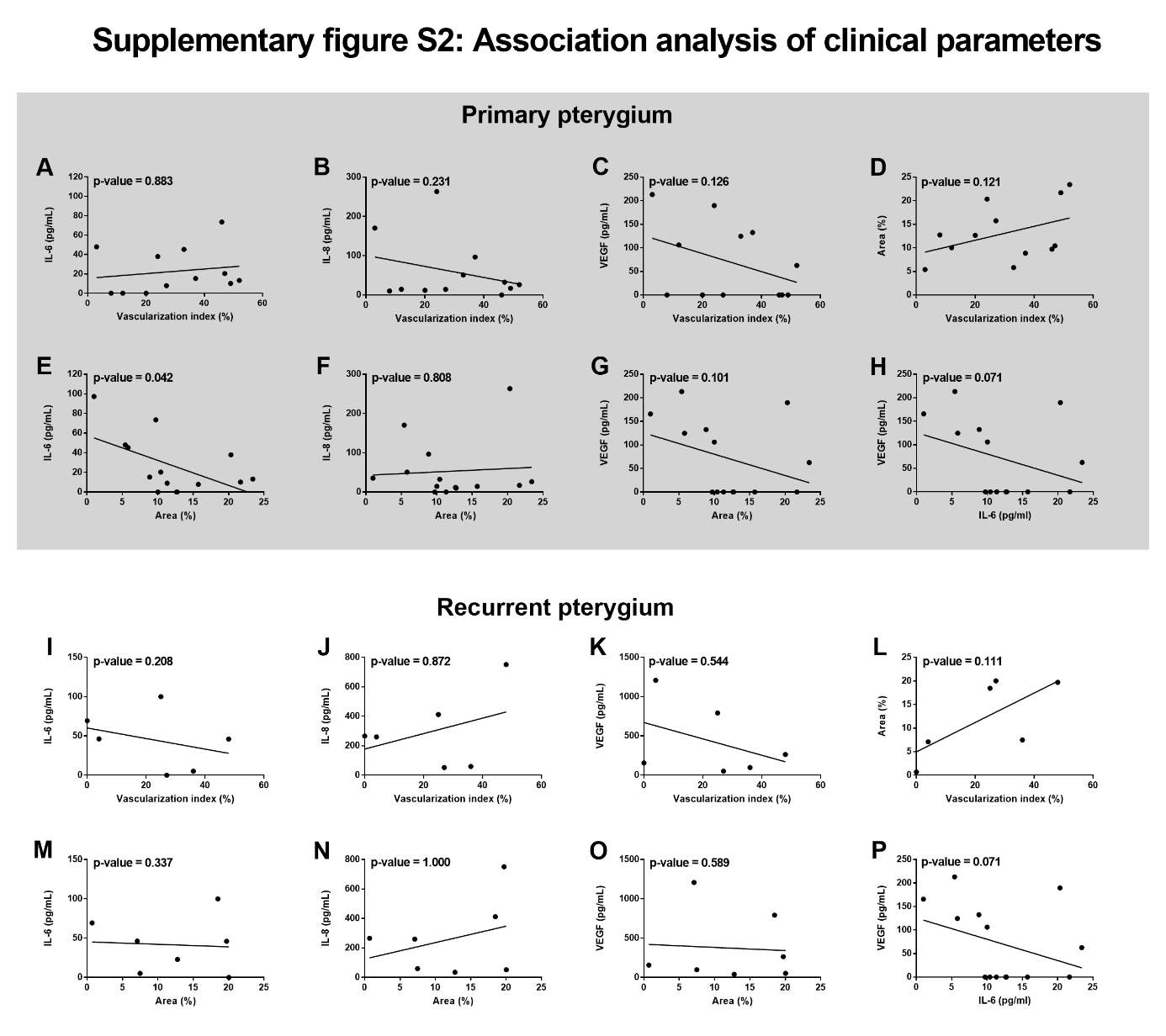

Supplement: Supplementary 2 — Supplementary figure S2: an overview of the association analysis between the preoperative (I) IL-6, IL-8, and VEGF tear film levels; (II) the overall vascularized area on the ocular surface (~vascularization index); and (III) the area on the cornea covered by pterygium (~area) in primary (S2A-H) and recurrent pterygium patients (S2I-P). [file 9416262.f2.docx]
