# Supplementary material for: Pterygium Pathology: A Prospective Case-Control Study on Tear Film Cytokine Levels
Source: Mediators Inflamm. 2019 Nov 12;2019:9416262. doi: 10.1155/2019/9416262 (PMC6875004; doi:10.1155/2019/9416262)
Supplement: Supplementary 3 — Supplementary table S3: the reported IL-6, IL-8, and VEGF tear film levels and the corresponding sampling method (i.e., tear type, location, and volume) in healthy individuals and patients with inflammatory ocular surface diseases. [file 9416262.f3.docx]

**Supplementary table S3**: IL-6, IL-8, and VEGF tear film levels in healthy controls and inflammatory ocular surface diseases

| **Control** | | | | **Ocular pathology** | | | | | **Method** | | | **Ref** |
| --- | --- | --- | --- | --- | --- | --- | --- | --- | --- | --- | --- | --- |
| **IL-6** | **IL-8** | **VEGF** | **Dev.** | **IL-6** | **IL-8** | **VEGF** | **Dev.** | **Pathology** | **Tear type** | **Location** | **Volume** |  |
| (mean, pg/mL) | | |  | (mean, pg/mL) | | |  |  |  |  | µL |  |
|  | - | - | - | 69.90 | - | - | - | Pterygium | RT |  | - | (Zidi et al., 2017) |
| - | 170.57 ± 22.90 | 575.05 ± 48.29 | SEM | - | 450.84 ± 94.53 | 249.59 ± 17.84 | SEM | Uveitis | NST | Lateral canthus | 4 | (Carreno et al., 2017) |
| 161.79 ± 150.37 | 573.88 ± 465.33 | 476.41 ± 286.34 | SD | - | - | - |  | - | - | - | 2-15 | (Martinez-de-la-Casa et al., 2017) |
| 13.43 ± 8.74 | - | - | SD | - | - | - |  | - | NST | Lower lid margin | 4-5 | (Wei et al., 2013) |
| 110 ± 142 | 572 ± 637 | - | SD | 366 ± 296 | 661 ± 550 | - | SD | Bacterial conjunctivitis | NST | Lower tear meniscus | 2.2-121,7 | (Fodor, 2009) |
|  |  |  |  | 109 ± 72 | 203 ± 170 | - | SD | Corneal foreign body |  |  |  |  |
| 42.1 ± 10.6 | - | - | SE | 88.6 ± 16.2 | - | - | SE | Sjörgen syndrome | - | Lower cul-de-sac | - | (Tishler et al., 1998) |
| 632.3 ± 167.9 | 16791.4 ± 2841.2 | - | SD | 1625.7 ± 430.9 | 48508.6 ± 9397.3 | - | SD | DED | NST | Lower lid, lateral canthus | 20 | (Massingale et al., 2009) |
| 130.4 ± 12.3 | 322.7 ± 33.5 | 2784.2 ± 328.4 | SEM | - | - | - |  | - | NST | Lateral canthus | 4 | (Carreno et al., 2010) |
| 35.2 ± 5.5 | 147.7 ± 15.8 | 2608.5 ± 249.8 | SEM | - | - | - |  | - | NST | Tear meniscus, lower lid | 5 | (LaFrance et al., 2008) |
| 31.7* | 283* | - | - | - | - | - |  | - | NST | Inferior tear meniscus |  | (Uchino et al., 2006a) |
| 3.59 ± 3.38 | - | - | Unk. | 24.99 ± 7.44 | - | - | Unk. | DED (Sjörgen syndrome) | NST | Inferior tear meniscus | 30 | (Yoon et al., 2007) |
|  |  |  |  | 13.7 ± 6.91 | - | - | Unk. | DED (Non-Sjörgen syndrome) |  |  |  |  |
| 26.5 ± 21.8 | 176 ± 72 | - | Unk. | 210.0 ± 282.9 | 1657 ± 2393 | - | Unk. | DTS w/MGD | NST | inferior tear meniscus | 30 | (Lam et al., 2009) |
| 13.9 ± 1.1 | 37.4 ± 8.7 | - | Unk. | 68.7 ± 107.9 | 259.1 ± 335.4 | - | Unk. | Conjunctivochalasis | NST | Inferolateral tear meniscus | 30 | (Erdogan-Poyraz et al., 2009) |
| 29.3* | 1084.3* | - |  | - | - | - |  | - | NST | inferior tear meniscus | 15 | (Uchino et al., 2006b) |
| 226.2 ± 29.6 | 731.4 ± 116.2 | - | Unk. | - | - | - |  | - | NST | Conjunctival cul-de-sac | - | (Nakamura et al., 1998) |

*median; DED, dry eye disease; DTS w/MGD, dysfunctional tear syndrome without Meibomian gland disease; RT, reflex tears; NST

**References**

Carreno, E., Enriquez-de-Salamanca, A., Teson, M., Garcia-Vazquez, C., Stern, M.E., Whitcup, S.M., et al. (2010). Cytokine and chemokine levels in tears from healthy subjects. *Acta Ophthalmol* 88(7)**,** e250-258. doi: 10.1111/j.1755-3768.2010.01978.x.

Carreno, E., Portero, A., Herreras, J.M., Garcia-Vazquez, C., Whitcup, S.M., Stern, M.E., et al. (2017). Cytokine and chemokine tear levels in patients with uveitis. *Acta Ophthalmol* 95(5)**,** e405-e414. doi: 10.1111/aos.13292.

Erdogan-Poyraz, C., Mocan, M.C., Bozkurt, B., Gariboglu, S., Irkec, M., and Orhan, M. (2009). Elevated tear interleukin-6 and interleukin-8 levels in patients with conjunctivochalasis. *Cornea* 28(2)**,** 189-193. doi: 10.1097/ICO.0b013e3181861d0c.

Fodor, M. (2009). *Cytokine detection in human tears in various anterior segment eye conditions (with special regard to penetrating keratoplasty).* Doctor of Philosophy, University of Debrecen.

LaFrance, M.W., Kehinde, L.E., and Fullard, R.J. (2008). Multiple cytokine analysis in human tears: an optimized procedure for cytometric bead-based assay. *Curr Eye Res* 33(7)**,** 525-544. doi: 10.1080/02713680802190085.

Lam, H., Bleiden, L., de Paiva, C.S., Farley, W., Stern, M.E., and Pflugfelder, S.C. (2009). Tear cytokine profiles in dysfunctional tear syndrome. *Am J Ophthalmol* 147(2)**,** 198-205 e191. doi: 10.1016/j.ajo.2008.08.032.

Martinez-de-la-Casa, J.M., Perez-Bartolome, F., Urcelay, E., Santiago, J.L., Moreno-Montanes, J., Arriola-Villalobos, P., et al. (2017). Tear cytokine profile of glaucoma patients treated with preservative-free or preserved latanoprost. *Ocul Surf* 15(4)**,** 723-729. doi: 10.1016/j.jtos.2017.03.004.

Massingale, M.L., Li, X., Vallabhajosyula, M., Chen, D., Wei, Y., and Asbell, P.A. (2009). Analysis of inflammatory cytokines in the tears of dry eye patients. *Cornea* 28(9)**,** 1023-1027. doi: 10.1097/ICO.0b013e3181a16578.

Nakamura, Y., Sotozono, C., and Kinoshita, S. (1998). Inflammatory cytokines in normal human tears. *Curr Eye Res* 17(6)**,** 673-676.

Tishler, M., Yaron, I., Geyer, O., Shirazi, I., Naftaliev, E., and Yaron, M. (1998). Elevated tear interleukin-6 levels in patients with Sjogren syndrome. *Ophthalmology* 105(12)**,** 2327-2329. doi: 10.1016/S0161-6420(98)91236-2.

Uchino, E., Sonoda, S., Kinukawa, N., and Sakamoto, T. (2006a). Alteration pattern of tear cytokines during the course of a day: diurnal rhythm analyzed by multicytokine assay. *Cytokine* 33(1)**,** 36-40. doi: 10.1016/j.cyto.2005.11.013.

Uchino, E., Sonoda, S., Nakao, K., and Sakamoto, T. (2006b). Alteration of tear cytokine balance by eye closure: analysis by multicytokine assay. *Graefes Arch Clin Exp Ophthalmol* 244(6)**,** 747-749. doi: 10.1007/s00417-005-0127-z.

Wei, Y., Gadaria-Rathod, N., Epstein, S., and Asbell, P. (2013). Tear cytokine profile as a noninvasive biomarker of inflammation for ocular surface diseases: standard operating procedures. *Invest Ophthalmol Vis Sci* 54(13)**,** 8327-8336. doi: 10.1167/iovs.13-12132.

Yoon, K.C., Jeong, I.Y., Park, Y.G., and Yang, S.Y. (2007). Interleukin-6 and tumor necrosis factor-alpha levels in tears of patients with dry eye syndrome. *Cornea* 26(4)**,** 431-437. doi: 10.1097/ICO.0b013e31803dcda2.

Zidi, S., Bediar-Boulaneb, F., Belguendouz, H., Belkhelfa, M., Medjeber, O., Laouar, O., et al. (2017). Local pro-inflammatory cytokine and nitric oxide responses are elevated in patients with pterygium. *Int J Immunopathol Pharmacol* 30(4)**,** 395-405. doi: 10.1177/0394632017742505.
